# The scale insects: Its status, biology, ecology and management in tea plantations

**DOI:** 10.3389/finsc.2022.1048299

**Published:** 2023-01-17

**Authors:** Beauti Kakoti, Bhabesh Deka, Somnath Roy, Azariah Babu

**Affiliations:** ^1^ Department of Entomology, Tocklai Tea Research Institute, Jorhat, Assam, India; ^2^ Department of Entomology, North Bengal Regional Research and Development (R & D) Centre, Nagrakata, West Bengal, India

**Keywords:** tea, pest, IPM, pesticide, Camellia sinensis, sooty moulds

## Abstract

The scale insects reduce plant photosynthetic ability by sucking sap from leaves and causing significant damage to the tea crop in most tea-producing countries. They suck the sap from stems and tea leaves, which not only prevents further growth but also reduces the nutritional quality of the leaves by promoting the growth of sooty molds. However, due to the widespread use of organosynthetic pesticides in recent decades, most insect pests have developed high levels of pesticide resistance, reducing the effectiveness of insecticide application. Bio-control agents are environmentally safe and produce long-term results while reducing the use of chemicals and other pesticides without disrupting the natural equilibrium. The review includes a list of coccidicides discovered on tea in major tea-growing countries as potential tea pests. The scope of future studies and the plans for better management of this serious sucking pest of the tea plant are also discussed in this review.

## 1 Introduction

**Figure 1:**
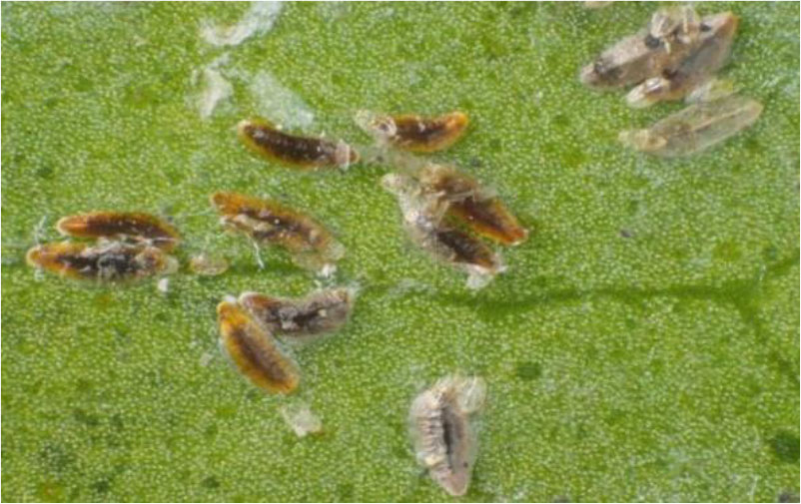
Tea scale insect *Fiorinia theae*.

**Figure 2:**
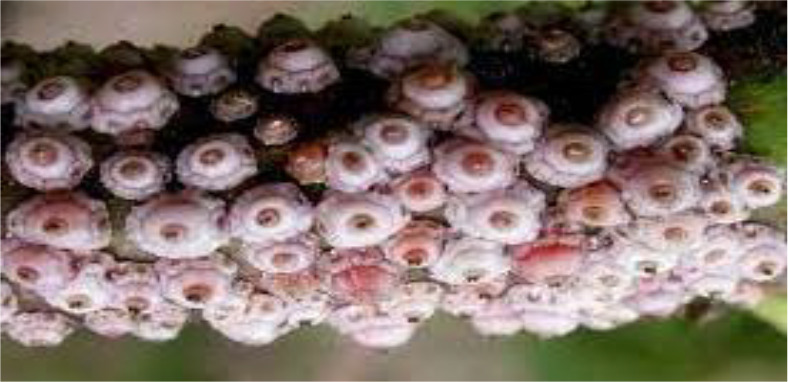
Tea scale insect *Ceroplastes rubens*.

**Figure 3:**
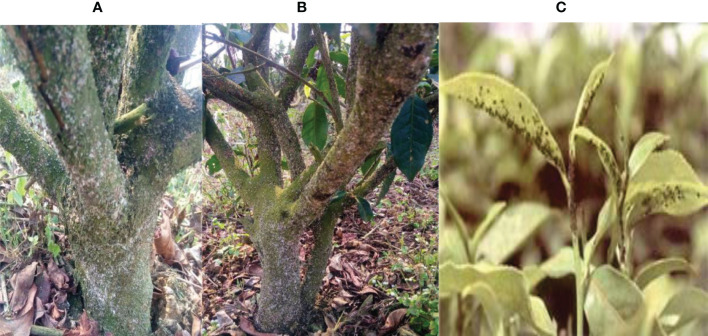
Infested tea plant by scale insect **(A-C)**.

The highly cultivated perennial monoculture crop, the tea plant, *Camellia sinensis* (L.) O. Kuntze, is grown on large- and small-scale plantations worldwide. A total of more than 50 nations now cultivate tea, which is sent from Georgia at 43 N latitude to Nelson in New Zealand’s South Island at 42 S latitude for consumption worldwide. China, India, Sri Lanka, and Kenya are the world’s top tea producers. The remainder of the world’s tea production is grown in Vietnam, Turkey, Indonesia, Argentina, Japan, Bangladesh, Malawi, Uganda, and Tanzania. The pest ecology of the tea plant is influenced by its particular traits. Especially in Southeast Asia, plantations of tea with genetically different cultivars and shade trees interplanted provide an evergreen and perennial (lasting over a century) product (1). A “single-species forest” may be seen in tea plantations, where insects and mites live by reducing competition *via* well-defined stratification and/or ecological niche development (2). Scale insects, like the other major tea pests, are polyphagous and sap-sucking insects. Scales and mealybugs are members of the Coccoidea superfamily. Scale insects are distinguished by the protective coating known as “tests” that are produced when eating. Several attractive plants in nurseries and landscapes, notably camellias and hollies, are affected by the tea scale, *Fiorinia theae* (Hemiptera: Diaspididae) (**Figure 1**). These pests reproduce rapidly and are difficult to eradicate due to the high number of overlapping generations they go through each year. Tea scale insects resemble aphids, white flies, and psyllids quite closely (3). Fiorinia theae and Ceroplastes rubens (**Figure 2**) cause the most damage to tea plantations in North East India and West Bengal. Toxic yellowing of the leaves caused by tea scales, which are coated scales, is a sign of a serious infestation (**Figure 3**). The plant keeps the damaged leaves for at least a season, even after control measures have been taken. One of the most destructive pests on tea plantations, the scale insect, has not been well studied. So, the goal of this review is to put together all of the information that is currently known about this pest’s taxonomy, bioecology, status, tolerance to pesticides, and other aspects of IPM programs, as well as future research that needs to be done to better control the scale insect on tea crops.

## 2 Taxonomy

The superfamily Coccoidea contains nearly 8000 species of plant-feeding hemipterans, comprising up to 32 families (4). Among all the families, members of the Coccidae (soft scales), Diaspididae (armoured scales), and Pseudococcidae (mealy bugs) are considered to cause most of the crop loss in the world (5). Armoured scales (Diaspididae) have the most species and subspecies in 371 genera and subgenera, with 2,383 species and subspecies, followed by Mealy bugs (Pseudococcidae) with 2,194 species. Coccidae is the third-largest family, with 1,281 described species in 176 genera (6), 146 of which are recognised as pests or potential pests globally (7). Coccoids differ in chromosome number, sperm structure (8), bacterial endosymbioses (9), and genetic systems, which include hermaphroditism, diplodiploidy, thelytoky, and haplodiploidy (10). Recent phylogenetic studies using molecular and morphological data support the recognition of up to 15 extant families of archaeococcoids, including 11 families. Molecular diagnostics as the basis for species identification is helpful because the approach is not limited by developmental stage or gender (11). Relationships among most scale insect families are unresolved in phylogenetic trees based on nuclear DNA sequences, and most nodes in trees based on morphological data, including those from adult males, are poorly supported. Within the Neococcoids, the Eriococcidae are not monophyletic, and the monophyly of the Coccidae and Diaspididae may be compromised by the current family-level recognition of a few species-poor autapomorphic groups (3). There are several species of scale insects observed in tea gardens in India, and the common ones are *Fiorinia theae* (Green) on tea leaves and *Ceroplastes rubens* (Mask ell) on tea stems.

## 3 Host range

Most scale insect species are host-plant specialists. But some species belong to the most polyphagous species known. For example, *Coccus hesperidium* feeds on plant species from at least 121 families (12). Scale insects in the tropics have larger host ranges (13). Coccids and diaspidids have been found on a wide variety of host plants, according to records. Almost 1181 coccid species have been found on a total of 1,993 species of host plants, 1,506 genera, and 240 plant families, whereas 2,624 species of diaspidids have been found on a total of 2,843 species, 2,043 genera, and 290 plant families. Fabaceae, Asteraceae, Rosaceae, Poaceae, Rubiaceae, Myrtaceae, Malvaceae, Moraceae, Rutaceae, and Sapindaceae are the ten most frequent host families for soft scales. Fabaceae, Poaceae, Rosaceae, Myrtaceae, Orchidaceae, Asteraceae, Euphorbiaceae, Pinaceae, Arecaceae, and Fagaceae are the ten most frequent host families for hard scales, listed from most common to least. Soft scales prefer woody perennials, whereas hard scales prefer long-lived plants such as trees and shrubs, although they may also be found on annuals (6, 14). Coccid species were found to be polyphagous on angiosperms in 37% of cases and gymnosperms in 48% of cases (15). Polyphagous coccids are a major problem for a number of key pest species. It was found that eight species of Coccidae were exceptionally polyphagous, eating plants from more than 50 different families, according to Lin et al. (15). One host genus or species may be attacked by multiple scale insect types because of its host plant setting. Coccid and diaspidid species have been found on citrus and mango, respectively, with 91 and 114 species of coccids on citrus and 68 and 70 on mango (6).

## 4 Damage caused by scale insects in tea

Soft scales are phloem-sucking insects (16). Scale insects feed on tea plant sap by piercing and sucking their mouth parts into the leaves. It is possible for scale insects to produce yellowing or drooping leaves, stunted or unappealing plants, and even plant mortality when infestations are high. Drought, insect pests, or disease may weaken weak plants, making them more vulnerable to damage and infection. Honeydew is a delicious, sticky fluid that soft-scale insects exude when they eat. Undigested sugar and water are combined to form honeydew, which the bug excretes onto the leaves and stems of plants (17). If the plant has honeydew, it may seem glossy or even moist, attracting insects such as bees and flies. Because of the honeydew, a fungus known as sooty mould may flourish, giving the plants a characteristically “sooty” black look. Scale insects usually do not occur in healthy, vigorous plants, and their presence is an indication of an imbalance of water and nutrients in the host (1).

## 5 Biology

The climate has an impact on the tea scale’s size and length. Adult tea scale females begin incubation four to six days before producing eggs when the temperature is between 86°F and 91°F (18). Underneath the armour, there are two rows of eggs. The “crawler” stage of the tea scale’s first instar nymph is a mobile stage that hatches in 10 days and is the sole stage in which an infestation may be transmitted. It takes them one to four days to crawl out from behind an adult female’s armour and begin searching for succulent plant tissues to bore holes in with the stylets they use to feed. After attaching their stylet, the crawlers will moult in about ten days.Sex can be determined after the first moult (19).Males are born yellow, but as they mature, they develop a thin, delicate white coat. It is at this phase that they will grow one pair of wings, one pair of halteres, and non-functional mouthparts before they achieve sexual maturity. It is the only function of the adult male to follow pheromones and mate with an adult mating female. Females require only two moults to reach sexual maturity.Six days after the first, the second moult occurs.The skin from the first moult will be retained by the female.Over time, the skin will harden and give the adult female her brown colour (20).

A fuzzy appearance may be caused by a dense concentration of crawlers, immature males and females, or even adults in extreme infestations. Temperature affects how long the tea scale takes to complete one cycle. After laying between 10 and 15 eggs, an adult female will begin to shrivel up and die. Scales can reproduce all year in warmer climates such as India, but hatching occurs more frequently when temperatures begin to rise in colder climates.

### 5.1 Eggs

Shiny golden, oval-shaped eggs are wide at one end and narrower at the other. The eggs become a dull yellow just before hatching. Ventral ducts and pores exude wax filaments that coat eggs. Each species has its own unique egg production rate, which is usually correlated with the size of a female’s body (21). Each female is capable of producing anywhere from a few dozen eggs up to several hundred or even thousands (16).

### 5.2 Crawlers

**Figure 4:**
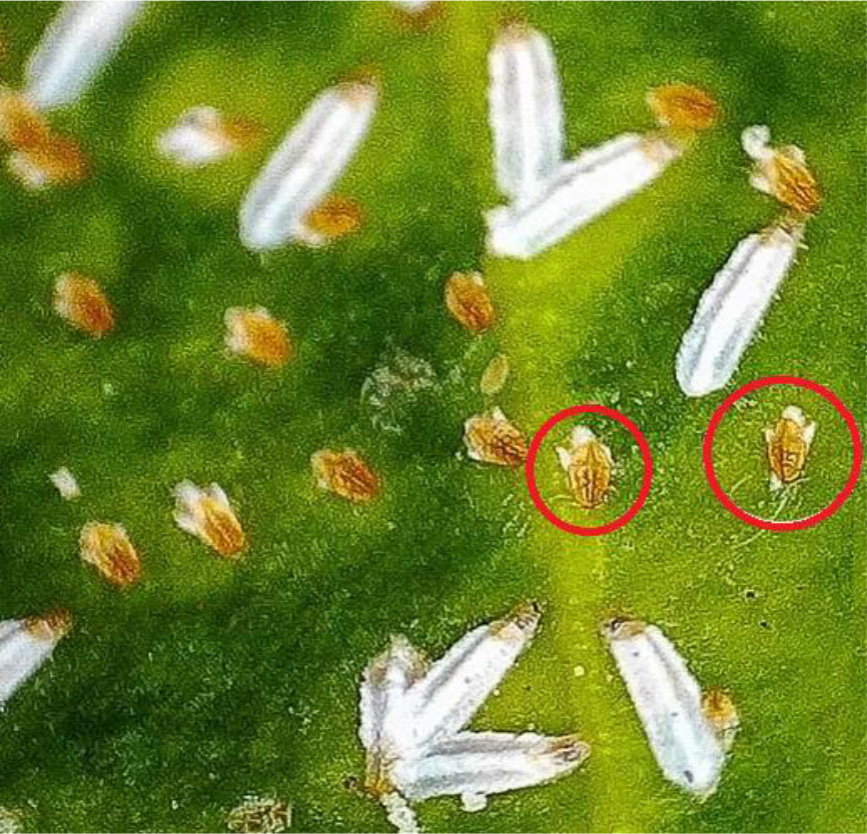
Crawlers of scale insects that just hatched from eggs.

First-instar nymphs hatch from the egg with fully developed legs and antennae, and they are the most active stage of the life cycle. First-instar nymphs are very small, less than 1 mm long and 0.5 mm wide, with an oval or elongated body (**Figure 4**). Koteja (22) was able to divide the life of a first-instar hard-scale nymph into four stages: postnatal torpidity, dispersal, feeding (growth), and morphogenetic (moulting). This distinction should also apply to first-instar soft-scale nymphs. The term “crawler” should strictly refer to the second period, which is the moving period of a first-instar nymph. Crawlers can move away from their mother either by crawling away or by being moved by the wind or phoresis (16).

Nymphs in their second instar are typically oval to round in shape. Their legs and antennae are either underdeveloped or fully developed (23). Most coccid species have third-instar nymphs (21). This stage is often very similar to the adult in appearance and can be easily overlooked or mistaken for a young adult female in the field; reliable recognition requires microscopical examination (23). Observations on the number of moults in the female life cycle of some species have been contradictory. Legs and antennae can be reduced or fully developed.

### 5.3 Adult female

The skin from the first moult remains on adult females, giving them a light yellow appearance.The skin will then harden and turn brown, making a narrow, long armour with a dark, clear ridge running down the middle. The adult female will still be yellow, but her cover will keep her from being seen.

Female soft scales go through four or five stages of development: egg, first, second, and, in most cases, third-instar nymph and adult. The adult female has no wings and is neotenic, resembling the nymphal stage (16). An adult female can continue to grow slightly or significantly after emerging from the previous instar, and her shape and colour change significantly prior to oviposition (16, 24). Adult female soft scales can grow to be two to eight times the size of the previous instar, becoming swollen and heavily sclerotized. As a result, the length of adult female soft scales of different species can range from 1 mm to 18 mm (24). Mature adult females lay their eggs in an ovisac enclosing, beneath, or behind the female body, or in a “brood chamber” beneath the female body (16, 24). The “brood chamber” is made when a space slowly forms under the abdomen (24).

### 5.4 Adult male

The adult male is orange-yellow in colour and has one pair of glossy forewings with fewer veins and a pair of halteres for back wings. As adults, males don’t have any mouth parts that work, so they follow pheromones to where females are waiting.

Coccid males have six life stages: eggs, first- and second-instar nymphs, pre-pupae, pupae, and adults. Eggs and first-instar nymphs are indistinguishable from those of females at the same stages. In second-instar nymphs, males are elongated and oval in shape and have tubular ducts on their bodies, whereas females are generally oval to round and do not have tubular ducts (23). Pre-pupae are surrounded by a semi-transparent glassy cover made up of plate-like structures produced by the second instar (25, 26). This stage marks the beginning of the body’s transformation into a form very different from that of a female. The body lengthens and becomes membranous, with shortened antennae and legs and the appearance of wing buds. Pupae are mostly membranous, but their antennae and legs become more sclerotized and longer, and their wing buds develop and extend more posteriorly (23). The adult male has well-developed front wings, antennae, and legs, as well as a well-defined head, thorax, and abdomen, with a noticeably elongated penial sheath at the abdomen’s tip (27). Congeneric variations make it difficult to generalise the biology of scale insects (28).

**Figure 5:**
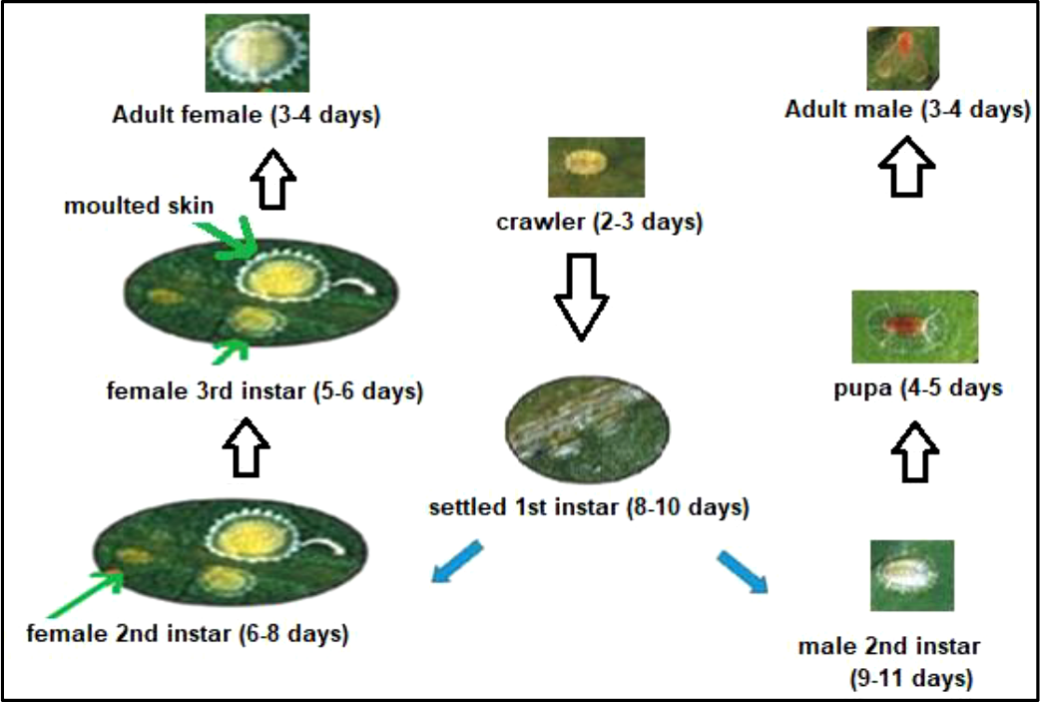
Life cycle of scale insect.

Reproduction can be either sexual or parthenogenetic. Females lay eggs under the protective covering called “Test.” Eggs hatch out into “crawlers” and are dispersed to new shoots by means of wind and attending ants sometimes (29). Females are neotenic and undergo 2–3 moults, attaining the non-winged adult stage by heterometabolous–parametabolous metamorphosis. On the other hand, males holometabolously metamorphose into winged adults. Most soft scales have one generation per year, with some exceptions. For example, the brown soft scales have multiple generations per year in warmer regions (30). The lifecycles of males and females are quite different (**Figure 5**; **Table 1**).

**Table 1:** Difference in life stages in male and female.

	Soft scale	Armoured scale	Mealy bug	References
Male	5	4	4	(30, 31)
Female	3	2	3	

Adult females live beneath their protective shield for their entire lives, while winged males fly around searching for females for copulation (28). Adult males have a very short life span as their functional mouthparts are lacking, unlike females. The first instar is called the “crawler” stage and is the most active (30) and dispersing stage (50). The second instar is sexually dimorphic, and in males, the second instar moults into a prepupal stage showing wing buds. Once dispersion has started, the crawlers settle on suitable feeding sites, and after cessation of feeding, the males can settle down on other host plants other than the mother plant and secrete the wax (68). Most soft scales move to the twigs of leaves to overwinter and come out in the spring. Exceptions can be seen in some soft scales; for example, females of *Ceroplastes* spp. overwinter in the adult stage and lay eggs early in the spring (30).

The body of the second-instar female is circular and oval in comparison to the male’s elongated oval body, and it can be distinguished from the male by the absence of the tubular duct on the dorsum. The third instar female is similar to the adult female, but has fewer setae and pores and does not develop wing buds like the third instar male (30).

A high level of sexual dimorphism can also be seen in armoured scales. Like soft scales, the adults of males, which have 5 instars, including two pupa-like quiescent stages, are winged, active, non-feeding, and short-lived; those of females, which have 3 instars, are morphologically reduced, non-motile, feeding, and significantly longer-lived (79). Fecundity rates vary by species, ranging from 24 eggs per capita for *Eucalymnatus tessellates* to 6,355 for Ceroplastes destructor (80).

## 6 Diversity and distribution

At present, about 8000 species of scale insects are known in the world. India accounts for 409 species under 166 genera belonging to 14 families and 14 subfamilies (81). The diversity and distribution of scale insects occurring on tea are presented in **Table 2**. A positive distribution of scale insects is found in almost all tea-growing countries (82). Green & Mann (83) studied coccoids occurring on tea plantations in India and Ceylon. In 1908, Green reported 102 species from India (45). 44 species of scale insects and mealy bugs have been recorded from tea in North-East India, of which only a few are *Eriochiton theae* (Green). *Pinnaspis theae* (Maskell), *Phenacaspis manni* (Green), etc., have been recorded to attain the status of major pests of mature tea bushes in the Darjeeling district. Lakshmishree et al. (84) studied the diversity of scale insects in the Tumakuru district of Karnataka in 2019 and reported 31 species belonging to 20 genera. 1,189 scale insect species belonging to 271 genera (15% of global distribution) have been recorded in China (85). There are over 204 different species of scale insects on the entire Korean Peninsula (6, 86). Takagi (72) listed 125 species of armoured scales in Taiwan on a wide range of hosts.

**Table 2:** Diversity and distribution of scale insects occurring on tea.

Genus	Species	Host range	Geographical distribution	Plant part infested	Generations per year	Report on Tea
*Ceroplastes*	*ceriferus*	Polyphagus; 60 plant families including *camellia sinensis Mangifera indica*; *Azadirachta indica* *Morus alba;* *Betulla, Salix, Platanus* and many other plants	India (12) New Guinea (32) Taiwan China (33) Japan (12) Sri Lanka (34)	Twigs, stems and branches (34)	1 (35)	China (33) Tokyo Korea
	*floridiensis*	Polyphagus; 70 plant families including *Camellia,Psidium*;*Pinushalepensis*;*Coffea arabica*; *Psidium pomiferum, Citrus* sp.; *Coffea Arabica* etc.	India (7, 34, 36) Mauritius (36) Sri lanka (34) Japan (12) Taiwan (12)	Leaves, stems, twigs and branches (34)	2 (34)	Taiwan North-east India (37) Sri lanka (34)
	*destructor*	Polyphagus*; Syzygiumcumini*, *Camellia sinensis*etc	India, New Guinea (34) Kenya (12) Uganda (12) Malawi,Africa (38)	Stem, twigs and branches (39)	1 (40)	South-africa (38) South pacific region.
	*japonicus*	Polyphagus with 36 host plant families. *Camellia japonica*, *Camellia sinensis*; *Camellia oleifera;Diospyros* spp.	China (41) Japan (12) USSR (42) Georgia (43)	Leaves and stem (42)	1 (44)	Georgia (43) Korea
	*pseudoceriferous*	*Azadirachta indica, Diospyros montana, Artocarpus heierophyllus*, *Ficus* spp.*, Psidium guajava, Madhuca indica, Croton* sp.*, Mangifera indica* etc.	Japan Taiwan Bangladesh (45) Sri Lanka (45) India (46) China (47)	Twigs (48)	3 (49)	India and Eastern asian Countries (50) Taiwan
	*sinensis*	*Camellia sinensis, Magnolia grandiflora, Citrus* sp.*, Cedrus deodara*	Iran (51). Turkey (52) Georgia (43)	Leaves	1	Georgia (43)
*Pulvinaria*	*floccifera*	*Camellia* spp., *Osmanthus fragrans*	Iran (53) Georgia (54) Virginia (55)	Leaves (56)	1 (57)	Iran (53) Georgia (54) Virginia (55)
*Coccus*	*discrepans*	*Camellia sinensis,banana, mango, Citrus* sp.*, Ziziphus mauritiana, Bauhinia* sp.*, Bignonio radicans, Dalbergia sissoo, Syzygium cumin;, Ficus carica, Morus alba etc.*	India, Sri Lanka, Pakistan (45)	Stem	—	North-east India (58)
	*viridis*		Sri lanka, India (58) etc.	Twigs	—	Sri Lanka(sarma, 32) North East India (58)
	*hesperidium*	*Camellia* sp. *Citrus* spp.*, Ficus sp, Magnifera* sp. *Morus etc.*	Uganda,Kenya,Tanzania & Ethiopia (12) India (7) Sri Lanka (34) Fiji (32) Kenya (58) Korea Japan (16)	Stem and leaf (7)	6 (59)	North east India (58) Fiji (32) Kenya (58) Korea Japan (16)
	*formicarii*	Highly polyphagus; on 46 plant genera in 31 families,including*avocado, guava,Garcinia, jackfruit, Japanese persimmon, mango, olive, Prunus, tea and velvet apple*	China, Ethiopia, India, Sri Lanka and Taiwan.	Stem	—	China (60) Sri Lanka.
*Lecanodiaspis*	*albilineata*	*Camellia* sp.	Neotropical: Guatemala (12)			Neotropical: Guatemala
*Saissetia*	*coffeae*	Highly polyphagus feeding on plant species from 313genera in 112families including coffeae, tea, guava, citrus etc.	Bangladesh, India, Sri Lanka (45) Japan (47) Papua New Guinea (40) Taiwan	Leaves, twigs, stem and fruits (61)	3 (62)	India (63)
	*oleae*	Highly poluphagus with host range upto 232 plant genera including *Camellia sinensis, Olea* spp.*, Citrus* spp.	China, India, Japan, Taiwan (12) e	Leaves and twigs (64)	2 (64)	India
	*nigra*	Polyphagus, feeding on plant species from 292 genera including *Citrus* spp. *Gossypium* sp.*, Morus* sp. *Camellia sinensis, Solanum* sp.*, Ficus* etc.	India,Sri Lanka, Taiwan, Thailand, and Vietnam (12). Bangladesh (45)	Leaves and Branches	1 (65)	North East India (58)
	*formicarii*	Tea plant*, Cinchona Sp.Macaranga* sp.*, Elaeocarpus* sp.	India and Sri lanka (45)	Stem	—	India (https://niphm.gov.in/IPMPackages/Tea.pdf, 32); Srilanka (50)
*Chloropulvinaria*	*psidii*	Polyphagus feeding on plant species in 158 generaincluding*Psidium, Ficus, Citrus,Mangifera, Morinda, Camellia, Coffea, Carissa,Eugenia, Litchi, Morus*	India, Sri Lanka, Bangladesh (34) China, Papua New gunia, Uganda, japan (12)		2 (66)	Papua New Guinea (32); Sri Lanka India (45)
	*floccifera*	Highly polyphagus on 122 plant genera including *Ficus, Olive, Coffae, Citrus* etc.	India Sri Lanka (34) Korea (67); New Zealand (68)	Leaves and stem (69)	1 (70)	Korea (71); New Zealand (68)
*Ceroplastodes*	*chiton*	*Cassia* sp.*, Cajanus,ajan, Solanum* sp.,tea plant*, Ficus* sp.*, Ziziphus mauritiana, Convolvulus* sp.*, Morus alba, Hibiscus syriacus*, etc.	India, Bangladesh, Sri Lanka (45)	Stems and small branches	—	India (58)
	*Cajani*	*Camellia, Ficus, Psidium* sp.etc	India and Sri Lanka (45)	Twigs	—	India (58)
*Dicyphococcus*	*castilloae*	*Vernonia* sp.*, Castilla elasticaAdenochlanazeyianica, Solanum* sp.*, Vernonia* sp., tea plantetc	India and sri Lanka (45)	—	—	India and sri Lanka (45)
*Eucalymnatus*	*tessellates*	Highly polyphagus on 122 plant genera including *Ficus, Olive, Coffee, Citrus* etc.	India (58) Sri Lanka (45)	Leaves (45)	1-2 (71)	India (58)
*Eriochiton*	*theae*	*Camellia* sp.	India (58), Sri Lanka (50)	Leaves and stem67)	—	India (58) Sri Lanka (50)
*Diaspididae*						
*Aonidiella*	*auranitii*	Highly polyphagus; including *Citrus spp, Capsicum, Camellia* sp.etc.	Taiwan (72) India (42)	Leaves and fruits (73)	Multiple (73)	India (58)
	*citrina*	*Camellia* sp.	USSR (57), Georgia (70)	Leaves		Georgia (70)
	*orientalis*	Highly polyphagus feeding on plant species of 176 genera including *Citrus* spp. *Ficus* sp.*, Morus* sp. etc.	India (42), Sri Lanka	Leaves	5 (74)	India (57). Sri Lanka
*Chrysomphallus*	*aonidium*	citrus, coconut, anthurium, bougainvillea, dendrobium, dracaena, eucalyptus, ficus, hibiscus, palm, ginger, *Citrus* spp, asparagus, tea, apple, mango etc.	Taiwan, Hawaii (74), India (50)	Stem and leaf	6 (17)	Japan (16) India (58) Taiwan (74)
	*pinnulifer*	polyphagus feeding on plant species of 61 genera including *Camellia sinensis, Magnifera indica, Jasminum, Psidium* etc.	India (75), African region	Leaves		India (42) African region
*Fiorinia*	*theae*	Polyphagus, feeding on plant species of 26 generaincluding*, Camellia sinensis, Citrus* spp. *Olea* sp. etc	Taiwan India (42)	Stem and leaf	Multiple (57)	United States (19) India (42)
*Pinnaspis*	*theae*	Plant species in 6 generaincluding *Camellia* sp.	India (42), Taiwan Japan and Sri Lanka (12)			China, India and Taiwan (42)
*Parlatoria*	*mytilaspiformis*	*Camellia* spp.	Taiwan (42), China (59),	Leaves		Taiwan (42), China (59)
*Hemiberlesia*	*rapax*	*Camellia sinensis* (76)	India (76)	Leaf axile (76)		West Bengal, india (76)
*Pseudococcidae*						
*Nipaecoccus*	*Viridis*	Highly polyphagus attacking plant species from 144 genera in 51 families including *Citrus, cotton, Euphorbia, Feronia, Morus, Camellia, Solanum, Psidium* etc	Bangladesh, India, Sri Lanka, China, Japan, Kenya and Uganda (77)	Leaves and stems		Himachal Pradesh, India (78)
*Pseudococcu*	*theaecola*	*Camellia* sp.	India (45) Sri Lanka (50)	Roots		India, Sri Lanka (58)
	*viburni*	*Camellia sinensis, Morus alba, Ficus* sp.*, Rosa sp, solanam* sp. *etc*,	Iran, China, Turkey (75)	Leaves		Iran (75) Southern Asia (63)
*Rastrococcus*	*ornatus*	*Jasminus* sp., Tea	India and Sri Lanka (45)	Foliage		India and Sri Lanka (45)
*Rhizoecus*	*theae*	*Camellia* sp.	India (50) Japan (42) Sri Lanka (50)	Root		Japan (42) India and Sri Lanka (58)

## 7 Dispersal

Crawlers are known to disperse actively by crawling away from their mother and/or passively through the effects of wind or phoresis (16). Newly emerged nymphs are dispersed up to several kilometers, mainly by the wind (87). Ants also help in dispersal by transporting and harbouring them in their nests.

## 8 Honeydew produced by soft scales

Soft scales feed on the phloem of the host plants and produce honeydew, while hard scales feed on cells of the mesophyll and do not produce honeydew (65). Honeydew is a sweet, sticky liquid excreted by soft-scale insects. It is a sugar- and water-based secretion that insects deposit on plant parts after passing through their digestive systems. Sugary, high-pressure liquid is released from the anus of the scale insects when their mouthparts pierce the phloem (88). Honeydew not only causes the plant to look wet and glistening, but it also attracts insects that feed on decaying matter, such as flies, ants, and bees (89).

### 8.1 Sooty mould

Among the saprophytic fungi known as sooty moulds, which form superficial black colonies on plants infested with honeydew-producing insects, are the soft scale insects (90). Sooty moulds are classified as ascomycete fungi in the order Dothideales, with five distinct family groups (90). *Antennariella, Aureobasidium, Capnodium, Cladosporium, Limacinula*, and *Scorias* are some of the most common genera of fungi that cause sooty moulds. Sooty moulds have a negative impact on plants, reducing photosynthesis and causing leaf and fruit drop, which in turn reduces crop yields. Some plant products are also affected by their appearance.

### 8.2 Soft scales and ants

Insects are protected from their natural enemies by the presence of ants, which increases their impact and damage on plants. Ant-soft scale relationships show three-way interactions between soft-scale ants and plants, which indicate both their positive and negative effects (91). Predators and parasitoids are protected, transportation is made easier, and diseases and unfavourable weather conditions are avoided thanks to the ant’s ability to remove honeydew (91). Ants, on the other hand, benefit from honeydew’s protein, lipid, and carbohydrate content. Ants’ obligate and non-obligatory attendance reduces honeydew contamination by scale insects (91). Ants are so important to several taxa of tropical and subtropical scale insects that they can only survive in the nest or shelter of an ant (89). These species have developed behavioural and morphological adaptations to coexist with ants, and this is evident in their natural habitats. Soft scales found inside the hollow chambers of “ant-plants,” like those found on coccid ants, have also been linked to the genus.

## 9 IPM strategies to control scale insects in tea ecosystem

The first level of knowledge needed for control is taxonomic or systematic information about the pest (92). About 7–15% of crop loss is attributed to pests, diseases, and weeds. Over several decades, synthetic chemical pesticides such as synthetic pyrethroid, endosulphan, quinalphos, and others have dominated pest control (93). Though chemical pesticides provide effective control, they are often associated with a number of negative side effects, including pesticide resistance, secondary pest outbreaks, harmful effects on human health and the environment (93, 94), and residual effects in tea (95). These problems have drawn attention towards the development of more eco-friendly alternatives that are biodegradable as well as effective (94).

These bio-pesticides, which are the secondary metabolites of plants such as terpenoids, alkaloids, and phenolics, are both environmentally friendly and cost-effective. Neem (*Azadirachta indica*) extract can be used for the management of scales. *Annona squamosa* contains lanolin and anona, which are useful against scales (96).

### 9.1 Monitoring

Insect pest monitoring is essential to IPM since it helps determine the best course of action to take against a given infestation and which control measure to use (97). To combat a specific insect pest infestation in a field at the ideal time while maximising control strategy and grower inputs, the intervention thresholds are a prime example (98). Early detection of scale insects is the most important step in management because it allows early detection of the pest. The sampling method is critical to successful management (53); Sampling procedures vary among crop systems. Scale insects are difficult to spot due to their small size and inconspicuous colouring. Damage to plants is often not noticeable until the population has grown to a certain size. Their numbers can be misinterpreted because their waxy exoskeletons adhere to leaves or bark, and dead scale does not usually fall off a plant soon after. The presence of crawlers can be determined by keeping an eye out for them on the leaves and branches or by setting up a modified sticky trap. The double-sided tape is used to create a sticky trap, or single-sided tape is wrapped around a branch or twig with the adhesive side outward (16). The search for honeydew and black mould is very important, as black mould is the first sign of a scale problem. Bark on trunks and major stems should be examined for scale if the entire plant is stunted, blossoms poorly, experiences patchy dieback, or displays any other signs of stress. Honeydew attracts ants, and they will defend it from potential enemies. Scale may be present if ants are still working on plants but no aphids are found.

### 9.2 Cultural practices

In the early stages of pest management, cultural controls are the first and most basic way to control the number of pests (99). Prevention of infestations and population control should be the key objectives of scale insect management in order to limit economic damage. Once established, scale insects may be impossible to eradicate because they are typically resistant to insecticides and/or actively shield their young. Scale insect populations may be kept under control with strong cultural management practises (100).

Proper application of fertilizers, trimming, and irrigation preserves plant health, encourages plant resistance to pest attack, and slows the population growth of sap-sucking insects (101). All stock should be carefully checked, especially host plant species that are likely to get scale insects (and other pests and diseases).It is important to check the roots as well as the rest of the plant for insect infestation. Propagation of plants using cuttings taken from plants that contain scale insects should be avoided. Carefully removing heavily infested plants may help limit the proliferation of scale insects. If the infestation is localised to a branch, that can be cut away.

After removing a shipment of plants infested with scale insects, cleaning up the area where they were grown (with bleach, farmcleanse, or a similar type of solution) and eliminating any remaining pests is essential (102); Crop debris and egg masses that have fallen from plants may have provided a safe harbour for scale insects for weeks. By cleaning and removing the infected plant materials from the green house and fields also help decrease the presence of any remaining pests or diseases from previous crop cycles (102). Field workers should avoid moving in areas known to be infested with pests such as scale insects. After leaving potentially infected regions, workers should disinfect their gear and uniforms. maintenance of a healthy growth environment through provision of an optimal growing environment and other necessities; weak plants are more vulnerable to pest damage at lower pest populations Elimination of any weeds that may have sprung up in the soil or nearby.

### 9.3 Chemical control

The Food Safety and Standards Authority of India (FSSAI), the European Union (EU), the Food and Agriculture Organization (FAO), the Codex Alimentarius Commission, and the U.S. Environmental Protection Agency (EPA) have all declared maximum residue levels (MRLs) in tea, limiting the pesticides that can be used for widespread application. Controlling tea pests has been suggested using a wide variety of pesticides, including but not limited to chlordane (10%) dust, 50% DDT W.P., Endrex (20%) EC, Gammexane (50%) W.P., 5% BHC dust, lindane (20%) EC, aldrin, dieldrin, and endrin (99). It was discovered that endosulfan, together with DDT and dieldrin, is an effective standard pesticide in the Dooars tea plantations in India (103). There was a ban on DDT usage in tea once endosulfan was introduced in northeast India. Endosulfan, monocrotophos, phosalone, Shalimar Tar Oil, dimethoate, fenitrothion, chlorpyriphos, and quinalphos are only some of the chemical insecticides that were authorised for tea pests in the 1970s (104). It was not until 1982–1983 that synthetic pyrethroids were first used in tea (Satyanarayana 1983). During 2000, insecticides such as endosulfan, quinilphos, phosphomidon, phosalone, acephate, dimethoate, chlorpyriphos, monocrotophos, oxydemeton methyl, lamda-cyhalothrin, beta-cyfluthrin, etofenprox, cartap hydrochloride, alpha-methyne, cypermethrine, and neem formulations were recommended for controlling tea pests (105). However, due to their lower maximum residue limits (MRL) values, only a few insecticides, such as deltamethrin, thiomethoxam, bifenthrin, profenofos, quinalphos, and thiacloprid, are now used to control tea insect pests (99).

Tea scales are often difficult to control because they infest the underside of the leaves, consistently reproduce during the warmer months, and have a waxy covering that resists chemical penetration. Spraying of ethion 50% EC 1ml/L gives significant control in severe infestations (106).

When used at low to moderate infestation levels, horticultural oils are efficient in reducing populations. Unfortunately, by the time a scale infestation is noticed, the damage has usually been done. Insecticides are most effective when used on crawling insects (107). Adding an adjuvant to the spray formulation and directing the spray toward the plant’s foliage may increase the effectiveness of chemical control. For maximum effectiveness, a contact pesticide should be applied as a foliar spray twice or three times at seven- to ten-day intervals (108). Dinotefuran is an efficient systemic insecticide, but it needs to be administered multiple times owing to its short residual activity. Spraying insecticide solutions containing clothianidin 50 WDG at 1:4500 (HV), thiamethoxam 25% WG at 1:4000 (HV), and quinalphos 25 EC at 1:400 (HV) with adjuvants such as agro-spray oil at a 0.5–1% concentration can provide effective control of scale insects in tea crops (109).

### 9.4 Biological control

Minimization of the use of chemical pesticides is of central concern in tea, and the oldest and most promising tactic is biological pest management (93). Several predator species, such as Coccinellid beetles and hymenopteran parasitoids, are known to be effective control measures against scales (**Table 3**). Besides, microbial control agents such as entomopathogenic viruses, bacteria, and fungi are considered to be good alternatives for controlling tea pests. Entomopathogens are better than predators and parasitoids because they are less likely to be hurt by insecticides (10).

**Table 3:** List of Biological agents for controlling scale insect.

Natural enemies	Type of biological agent	Family	Target pest species	Reference
*Diversinervus elegans*	Parasitoid	*Encyrtidae*	*Saissetiaoleae*	(110)
*Metaphycus flavus*	Parasitoid	*Encyrtidae*	*Saissetiaoleae, Coccus hesperidium*	(110)
*Metaphycuszebratu*	Parasitoid	*Encyrtidae*	*Saissetiaoleae*	(110)
*Scutellistacaerulea*	Parasitoid	*Pteromalidae*	*Saissetiaoleae, Coccus hesperidium*	(110)
*Marietta leopardina*	Parasitoid	Aphelinidae	*Saissetiaoleae*	(110)
*Aphytis* spp.	Parasitoid	Aphelinidae	*Aonidellaaurantii, Abgrallaspiscyanophylli, Chrysomphalusdictyospermi, Fiorinia theae*	(110)
Coccophagus spp.	Parasitoid	Aphelinidae	*Coccus hesperidium*,	(110)
*Diversinervus elegans*	Parasitoid	Encyrtidae	*Coccus hesperidium*	(110)
*Alaptuspallidicornis*	Parasitoid	Mymaridae	*Coccus hesperidium*	(110)
*Azyaorbigera*	Predator	Coccinellidae	*Aonidiella oriental, Coccus viridis*	(111)
*Chilococcus* spp.	Predator	Coccinellidae	*Chrysomphalusaonidium, Aonidellaorientalis, A. auranti*	(111)
*Cryptolaemus montrouzieri*	Predator	Coccinellidae	*Chloropulvinariapsidii, planococcuscitri*	(111)
*Exochomusquadripustulatus*	Predator	Coccinellidae	*Saissetiaoleae*	(111)
*Nephus* spp.	Predator	Coccinellidae	*Planococcuscitri*	(111)
*Serangiumparcesetosum*	Predator	Coccinellidae	*Coccus hesperidium*	(111)
*Ankylopteryx* sp.	Predator	Neuropterida	*Pulvinaria* sp.	(7)
*Ceraeochrysa* spp.	Predator	Neuropterida	*Fiorinia theae, Chrysomphalusaonidium, Planococcuscitri*	(7)
*Chrysopa* spp.	Predator	Neuropterida	*Planococcuscitri,Coccusviridis, Saissetiaoleae*	(7)
*Coccotheraspissana*	Predator	Totricidae	*Ceroplastes* spp.	(112)
*Synanthedoncoccidivora*	Predator	Sessidae	*Ceroplastes* spp.	(113)
*Spalgisepins*	Predator	Lycanidae	*Eriochiton theae*	(114)
*Alternaria* spp.	Entomopathogenic fungi	Pleosporaceae	*Aonidella aurantii, Chrysomphalus ficus, Pulvinaria* sp.	(115)
*Aspergillus flavus*	Entomopathogenic fungi	Trichomaceae	*Pulvinaria* spp.	(115)
*Beauveria bassiana*	Entomopathogenic fungi	Cordicipitaceae	*Pulvinaria psidii*	(115)
*Metarhizium anisopliae*	Entomopathogenic fungi	Clavicipitaceae	*Pulvinaria citri, Planococcuspsidii*	(115)
*Septobasidium bogoriensis*	Entomopathogenic fungi	Septobasidiaceae	*Eriochiton theae*	(35)
*Verticillium lecanii*	Entomopathogenic fungi	Cordycipitaceae	*Ceroplastes destructor*	(35)
*Fusarium* spp.	Entomopathogenic fungi	Nectriaceae	*Ceroplastes sinensis*	(35)
*Serratia marcescens*	Entomopathogenic bacteria	Yersiniaceae	*Chrysomphallusficus*	(116)

## 10 Future prospects for effective management of the scale insects

Studies that have already been done show that the ways we deal with the scale insects that hurt tea plants could be better than they are now. Growers routinely use pesticides, which may result in resistance, resurgence, replacement, and residual problems. To combat this, effective and responsible use of all agroecosystem components is essential. IPM is an ecosystem-based strategy to pest management that prioritises long-term pest suppression using several methods, including but not limited to cultural, biological, biotechnological, and natural habitat alterations. To ensure that the targeted pests are controlled without causing damage to the environment or organisms that are not the intended targets, the use of chemical pesticides should be carefully considered, followed by careful monitoring, and supervised by strict regulations. Potential pesticides against scale insects should be tested for their ability to inhibit the function of specific biochemical sites, such as those involved in the synthesis of juvenile hormone (JH) biosynthetic enzymes, G-protein-coupled receptors (GPCRs), and transcription factors from the basic Helix-Loop-Helix (bHLH) family. Implementing integrated biological control strategies, which combine augmentation and conservation biological control with habitat manipulation, may provide effective alternatives to chemical-based pest management. The efficiency of biological control measures is dependent on the ability of natural enemies to manage pest problems as well as their ability to survive and spread throughout the ecosystem. Governments or funding agencies in tea-producing nations should provide adequate resources for scale insect research. To effectively execute the IPM programme, novel approaches, such as those discussed in this review, and technology transfer are required.

## 11 Conclusion

A list of coccids found on tea in major tea-growing countries as potential tea pests is included in the review. They suck the sap from the tea leaves, which not only prevents further growth but also reduces the nutritional value of the leaves by encouraging the growth of sooty moulds. Tea production requires close monitoring of pest incidence and proper implementation of IPM practices. Insecticide sprays have become the most common method of controlling insect pests in recent years. However, due to the widespread use of organo-synthetic pesticides in recent decades, most insect pests have developed high levels of pesticide resistance, reducing the effectiveness of insecticide application. Pesticides also kill beneficial insects and reduce the quality of tea leaves, negatively impacting human health. As a result, biological control has recently received a lot of attention as an alternative control mechanism that is non-hazardous to the environment and produces long-term results while reducing the use of chemicals and other pesticides without disrupting the natural balance. So, framers should use biological control and only use a small amount of insecticide to stop pest infestations.

## Author contributions

BK prepared the draft manuscript, BD edited and prepared the final manuscript, and SR and AB guided during the preparation of the manuscript. All authors contributed to the article and approved the submitted version.
